# Supplementary material for: A Lexicon of Descriptive Sensory Terms for Peas (Pisum sativum L.): A Systematic Review
Source: Foods. 2024 Jul 20;13(14):2290. doi: 10.3390/foods13142290 (PMC11276475; doi:10.3390/foods13142290)
Supplement: Supplementary file 1 [file foods-13-02290-s001.zip › foods-3079637-supplementary.pdf]

Supplementary Material.

Overview of selected sources of evidence:

The first shortlisted study [A] fulfilled the review's inclusion criteria as 5 descriptive sensory terms for peas were presented. Those were: *raw-beans*, *grassy*, *milk-like*, *earthy* and *fatty*. The sensory analysis was carried out using pea cultivars from China, which were processed into pea-milk, and tested by a sensory panel of 12, 4-males and 8-females aged between 25-32. The panellists were asked to list all the sensory attributes for the prepared samples during sensory training. 5 descriptive terms were the most popular attributes which have been shortlisted from the subsequent sensory evaluation. The study did not include information on the type of applied descriptive sensory terms which were under investigation, however, based on the study's results, it can be assumed that the listed descriptive sensory terms have been developed through olfactory and gustatory testing, making these 'flavour' based, see table 2.

The second study [B] contains information on experiments with pea flour milled from raw peas. Those were cultivated in the years 2019 and 2020 in China. The sensory attributes of the flour were tested using both, instrumental and panel-based evaluation techniques. The instrumental investigation was mainly carried out using G-C, where the volatile compounds present in roasted pea-flour were identified and analysed using an olfactory detection port equipped with odorant detection capabilities. The applied descriptive sensory terms detected through that approach were classed as 'odorants', 27 in number, see table 2 for the exact key words. Simultaneously, the sensory panel evaluation was carried out with trained panellists, 10 in number, 4-males and 6-females, aged 23-38. The panellists have identified and used 7 descriptive sensory terms for the evaluation, as follows: *grass-like*, *beans-like*, *fatty*, *popcorn-like*, *nutty*, *potato-like*, and *smoky*.

The instrumental analysis' results were the subject to further literature review to clarify their eligibility. This was due to some limitations found in the stated methodologies. Instrumental sensory analysis lacks the human subjectivity element and could not be suitable for preference testing, however it has been decided that the detected odorants and compounds through the instrumental analysis related to the existing aromas/flavours, which are in fact recognizable by the human olfactory and gustatory senses through which smell, and taste are perceived, and could form basis for panel-based preference testing [23]. As a result of that, the 27 odorants were included in the list identified through the review as fulfilled the descriptive sensory terms criteria.

Study [C] was based on the texture analysis of thawed peas. The evaluation was carried out in the United States with 11 panellists, 5-male and 6-female, where age was not declared. The panel was trained on the detection of texture for legume-like foods. In the article, the panellists had previously established definitions of the descriptive sensory terms, (potentially sourced from external literature). The study has not specified whether the samples were *Pisum sativum* L. or other 'pea' like legumes. As a result, the study had to be reassessed for eligibility, however, it was found to fulfil the inclusion criteria as the applied descriptive sensory terms that were investigated matched the attributes of *Pisum sativum* L. Those were the texture attributes, as follows: *crispness*, *juiciness*, *seed hardness*, *mealiness* and *Testa toughness*. All those texture attributes are present in most legumes, including *Pisum sativum* L., therefore, the terminology could be reapplied to peas, therefore, the study was included for data extraction [22-24].

Study [D] was performed using 12 different cultivars of *Pisum sativum* L. The study has defined the cultivars as domesticated and semi-domesticated varieties of peas. Pea protein isolate was extracted

from the selected cultivars using the alkaline extraction with isoelectric precipitation method. The Pea-protein isolate was served in 20ml sized samples to a semi-trained sensory panel. The study did not specify the number of panellists and whether the applied descriptive sensory terms were developed by the panellists themselves or if were borrowed from existing literature. The sensory attributes used for the sensory evaluation of the pea-protein were as follows: *fatty*, *green*, *earthy*, *roasty*, *pea-like*, *metallic*, malty, *nutty*, *bitter*, *sweet*, *salty*, *astringent* and *mouth-coating*. Due to a lack of definitions in the original study, these attributes have been classified under 'flavour'. The classification criteria were determined by the nature of the words, inflicting on their flavour-like characterisation [22,23].

Study [E] included results from a descriptive sensory evaluation of 6 frozen green peas (*Pisum Sativum* L.) purchased in wholesale and retail shops in South Africa. 12 trained panellists, 10-females, and 2-males, where age was not declared. The panellists have come up with a comprehensive list of sensory attributes counting 27 descriptors (see table 2), for appearance/visual, flavour and texture of the selected frozen garden peas. Definitions were also developed by the panellists and used throughout the sensory evaluation. Some of the sensory attributes were labelled with the words 'taste' and 'odour' but were finally classified as 'flavour'. A similar classification approach has been adapted for the purpose of this review, as a result, all of the applied descriptive sensory terms identified from this study were grouped under the 'flavour' category.

The authors of study [F] have conducted an extensive systematic review of literature for the "off-flavours" for various pulses, including peas. The off flavours have been defined as those that are released throughout the life cycle of the pulse, from farm to fork. Furthermore, some of those off-flavours have been associated with the product going past its use-by-date. This study has been included in the review due to its relevance to the topic with the descriptive sensory terms that were identified, 14 in number, being added to table 2. The authors have stated that the tested peas were taxonomically belonging to *Pisum sativum* L. The number of sources included in the systematic literature review has not been stated.

In study [G] pea (*Pisum sativum* L.) and soybean cultivars from Poland were used. The study's objectives were to identify, classify and evaluate the applied descriptive sensory terms for 'legume sprouts'. The pea sprouts were prepared in 2 different ways, first included a 3-day germination under UV lights in an incubator, and the second sample was incubated without the UV lightning. The nine-member trained sensory panel, aged 24 to 50 (no gender information) was tasked with the evaluation of the sensory profiles of the pea sprouts according to an earlier self-prepared list of sensory attributes, 13 in number (see reference G in table 2). The list of sensory attributes also included definitions for each, which allowed the panellists to identify the necessary descriptive sensory terms when scoring their strength and intensity. This study was determined as eligible as it was carried out in accordance with the principles of sensory evaluation of foods [23, 24].

Study [H] was carried out using Swedish landraces and cultivars of various fruits and vegetables, of which 6 were peas (*Pisum sativum* L.). The peas were first dried, frozen, and stored for several days, and then boiled and served as whole. The sensory evaluation was carried out by an untrained panel of 26 university students. The sensory evaluation participants were to test the samples using olfactory and gustatory senses and report on all the notable descriptive sensory terms. Resulting in a comprehensive list of applied descriptive sensory terms of which only 9 were selected for further evaluation and finally included in the paper, those were: (1) *earthy*, *odour intensity* (odour and taste), (2), *taste intensity*, *sweet*, *nutty*, *bitter* (taste) and (3) *dry*, *mealy* and *chewing resistance* (texture), see table 2 for the list of keywords. The selection criteria within the study were based on the intensity of

hints, in other words, only the sensory attributes that were most frequently detected by the sensory panel were shortlisted.

Study [I] focused on five commercial pea (*Pisum sativum* L.) preparations (powders) from different producers which were analysed including three isolates (PPI A, PPI B, PPI C), one concentrate, and one flour with over 120 volatile sensory compounds identified. These were analysed instrumentally and have therefore not been included in the findings of this review (as no descriptive sensory terms have been provided). Additionally to that, a trained panel has been used (1 male, 12 females, age 22–44 years) to evaluate the aroma attributes of pea protein preparations that have been evaluated through odour active solutions derived from pea products. The study has reported on 13 descriptive sensory terms that have been identified by the panel, either as smell (orthonasal/retronasal, n = 11) or as taste (n = 2). Those 13 descriptive sensory terms have been derived through methods fulfilling inclusions criteria of this review. Some of these terms have been presented as two-word lexemes, such as *cucumber and green* or *honey-like and sweet.* Those have been added to table 2 results as individual entries and not as separate descriptors as this is how those terms have been portrayed by the original authors of the study.

Study [J} was based on a field pea (*Pisum sativum* subsp. *arvense* L.) water based, fermented extracts in Italy. Visual, taste and olfactory (smell/odour) characteristics were measured by 10 untrained panellists (further detail is not available) over 28 days at 7-day intervals. The sensory panel has identified 7 different descriptive sensory terms, 2 of which have been reported by the authors to origin from their initial literature review. All 7 descriptive sensory terms have been added to results presented in table 2. It is worth noting that the intensity of these different attributes changed over time as the products were undergoing lactic-acid fermentation, however, the terms themselves have not changed over time.

Study [K] was based on German pea cultivar *Navarro* of *Pisum saitvum* L., which was used to produce pea protein isolates which were then fermented and tested for sensory attributes. 8 trained panellists (further detail not provided) have evaluated water-based samples for specific aroma (odour) and taste characteristics. Overall, the panel has identified 13 descriptive sensory attributes (11 odour and 2 taste). Those have been derived through methodological approaches fulfilling the inclusions criteria of this review and have therefore been added to the results as presented in table 2. It is important to note that those sensory characteristics have been based on fermented products (similar to that of study J) thus might not be applicable to all pea products nor fresh peas.

Study [l] was based on 6 Swedish and Danish landraces of peas (*Pisum sativum* L.) that have been cultivated in their natural environmental settings across the two countries. Peas were first soaked, then cooked *sous-vide* and served at room temperature. Overall, 10 undergraduate university students (trained) have taken part in the sensory analysis. The panel was trained using a CATA method which consisted of over 46 pre-existing terms (literature based), 27 new descriptive terms that were developed by the panellists during training and a final list of 20 descriptive sensory terms (9 for odour and 11 for taste) that have been used for the final sensory evaluation. The final 20 are from the within the list of terms developed during training but are differentiated by odour or taste and have therefore been groups accordingly to these criteria in table 2. The remining terms (initial CATA training and new terms developed by panel during training) have also been added to the results table but under flavour category, as no definitions have been provided but the methodological overview suggests that standard gustatory/olfactory testing has been conducted during the training. In summary, 72 descriptive sensory terms have been added under the flavour category and 20 have been added either as odour or taste. No definitions of the different descriptive terms have been provided by the authors.
